# The alarmin IL33 marks a thymocyte dependent shift in the makeup of cortical thymic epithelial cells

**DOI:** 10.3389/fimmu.2026.1974594

**Published:** 2026-10-27

**Authors:** Andrea J. White, Sonia M. Parnell, Fabrina M. Gaspal, Andrea Bacon, Nahim A. Nurjahan, William E. Jenkinson, Graham Anderson, Kieran D. James

**Affiliations:** Department of Immunology and Immunotherapy, School of Infection, Inflammation and Immunology, College of Medicine and Health, University of Birmingham, Birmingham, United Kingdom

**Keywords:** cTEC, IL33, thymic epithelial cell, thymocyte, thymus

## Abstract

Functional competence in the adaptive immune system requires the generation of αβT-cells in the thymus, where thymic epithelial cells (TEC) drive thymocyte development. Roles for cortical thymic epithelial cells (cTEC) include the generation of CD4^+^CD8^+^ thymocytes, and positive selection of cells capable of self-peptide/MHC recognition. Recently, we showed that, in the adult thymus, cTEC consist of two distinct subsets, Foxn1^+^ typical and Foxn1^-^ atypical cells. While typical cTEC regulate thymopoiesis through their expression of *Psmb11*, *Cxcl12* and *Dll4*, the developmental requirements and properties of atypical cTEC remain undefined. Here, we show atypical cTEC downregulate the expression of multiple genes controlling antigen processing and presentation for positive selection including *Psmb11*, *Cd83*, *Prss16*, and *Ctsl*. In addition, we show atypical cTEC acquire a distinct gene profile including the chemokines *Ccl9* and *Ccl11* together with the alarmin *Il33* that plays an essential role in thymic repair following injury. Using precursor-product analysis and *Cxcl12/Il33* dual reporter mice, we show *Il33*-expressing atypical cTEC can be generated from typical cTEC that lack *Il33* expression, a process which depends upon progression beyond the pre-TCR-mediated checkpoint. Together, these data define thymocyte-dependent changes in the adult cTEC compartment and suggest atypical cTEC may acquire functional properties that are distinct from their typical cTEC counterparts, including the possibility of acting as an additional source of IL33 in pathways of thymic regeneration.

## Introduction

In the thymus, immature thymocytes undergo multiple developmentally regulated events that result in the generation of functionally competent CD4^+^ and CD8^+^ T-cells. As thymocyte development is a non-cell autonomous process, interactions with cells of the surrounding stromal thymic microenvironment are essential to ensure a correct developmental program takes place. Thymic stromal cells are heterogenous, consisting predominantly of epithelial cells and mesenchymal cells, each containing significant heterogeneity (1–3). At a functional level, this remains only partly understood. In addition, the anatomical organization of the thymus into outer cortical and medullary areas has functional significance, with early stages in thymocyte development including the generation, and positive selection, of CD4^+^CD8^+^ thymocytes occurring in cortical areas, and intrathymic tolerance and post-selection maturation occurring in medullary areas (4, 5).

Of particular importance for thymus function are thymic epithelial cells (TEC). For example, in the thymic cortex, cortical thymic epithelial cells (cTEC) represent key regulators of multiple stages of thymocyte development (6, 7). During early stages of thymocyte development, cTEC act as a source of the chemokines CXCL12 and CCL25 to aid the entry of T-cell progenitors into the thymus (4, 8–10). In addition, the maturation of CD4^-^CD8^-^ thymocytes that have successfully rearranged TCRβ genes and express the pre-TCR complex requires Notch signaling which occurs via cTEC expression of DLL4 (11, 12). Finally, for positive selection of CD4^+^CD8^+^ thymocytes, cTEC express several molecules that operate together with their surface expression of both MHC class I and MHC class II to support the production of self-MHC restricted single positive (SP) thymocytes (13). For CD4^+^ positive selection, these include genes encoding Cathepsin-L (14), Prss16 (15) and CD83 (16, 17), while β5t - a component of the thymoproteasome encoded by the *Psmb11* gene that is uniquely expressed by cTEC - is important in generating an MHC class I bound self-peptide repertoire for CD8^+^ positive selection (18–20). Collectively, these findings show cTEC express, and are defined by, a gene profile that enables them to support thymocyte development. That many of these cTEC-expressed genes are also targets of Foxn1 (21), the master regulator of TEC development, further underpins the importance of this transcription factor in thymus development and function.

Many recent studies have highlighted significant heterogeneity within the TEC compartment, and this is perhaps most extensively studied within the mTEC lineage (22–24). For example, MHC II^hi^CD80^hi^ mTEC contain cells that express Aire and are important for tolerance induction (25) while MHC II^lo^CD80^lo^ mTEC contain both post-Aire mTEC mimetics that also influence tolerance mechanisms (26–28), as well as progenitors that maintain the mTEC lineage (29, 30). While our understanding of cTEC heterogeneity and its possible significance is less well developed, several studies have identified multiple subsets within the cTEC lineage (6, 31, 32). In our own recent work, we showed that adult cTEC, defined as EpCAM1^+^Ly51^+^UEA-1^-^, consists of two distinct compartments. First, we defined a Foxn1-expressing subset we termed typical cTEC, which express many of the known Foxn1-dependent regulators of thymopoiesis including *Cxcl12* and *Dll4*. In addition, we also reported a subset of EpCAM1^+^Ly51^+^UEA-1^-^ adult cTEC that had lost expression of Foxn1 and its downstream targets (31). As the properties and possible functional relevance of these atypical cTEC remains unclear, in this study we have analyzed multiple aspects of atypical cTEC biology, including their transcriptomic profile alongside their developmental origins and requirements. We propose that rather than representing a moribund post-Foxn1 subset, atypical cTEC are endowed with a thymocyte-controlled gene expression signature and may be capable of influencing both thymus regeneration pathways and the migration of multiple immune cell populations.

## Results

### Loss of a thymopoiesis-inducing gene signature in atypical cTEC is accompanied by the acquisition of multiple regulators of innate immune cells

A key feature of the thymic cortex is its ability to support the positive selection of immature CD4^+^CD8^+^ thymocytes that express αβT-cell receptors (αβTCRs) recognizing self-peptides bound to either MHC class I (MHC I) or class II (MHC II) molecules (6). In our previous work, we identified two distinct subsets of EpCAM1^+^Ly51^+^UEA-1^-^ cTEC in the adult thymus, which we termed Foxn1^+^ typical and Foxn1^-^ atypical cTEC (31). In that study, we used a mouse model where the fluorescent protein DsRed reported expression of *Cxcl12*, a known Foxn1-dependent gene, such that Foxn1^+^ typical cTEC are *Cxcl21^DsRed+^*, while Foxn-1^-^ atypical cTEC are *Cxcl12^DsRed-^* (31). To examine expression of key regulators of cTEC function in typical and atypical cTEC, we reanalyzed our previously published bulk RNA sequencing dataset (GSE205940) (31) from FACS-sorted DsRed^+^ typical and DsRed^-^ atypical EpCAM1^+^Ly51^+^UEA-1^-^ cTEC isolated from adult *Cxcl12*^DsRed^ reporter mice (31). In line with our previous work, we found that genes associated with T-cell development, including *Cxcl12 and Dll4* (9, 11, 12) were restricted to the typical *Cxcl12^DsRed+^* cTEC subset (**Figure 1A**). Interestingly, this differential pattern of expression also includes *Cd83* (16, 17)*, Psmb11* (18–20), and *Prss16* (15) (**Figure 1A**), that were previously identified as Foxn1-dependent genes (21) and represent important regulators of cTEC antigen presentation/processing. Thus, key genes for positive selection are differentially expressed in typical versus atypical cTEC, suggesting that the ability to support positive selection may be limited to some, but not all, adult cTEC.

**Figure 1:**
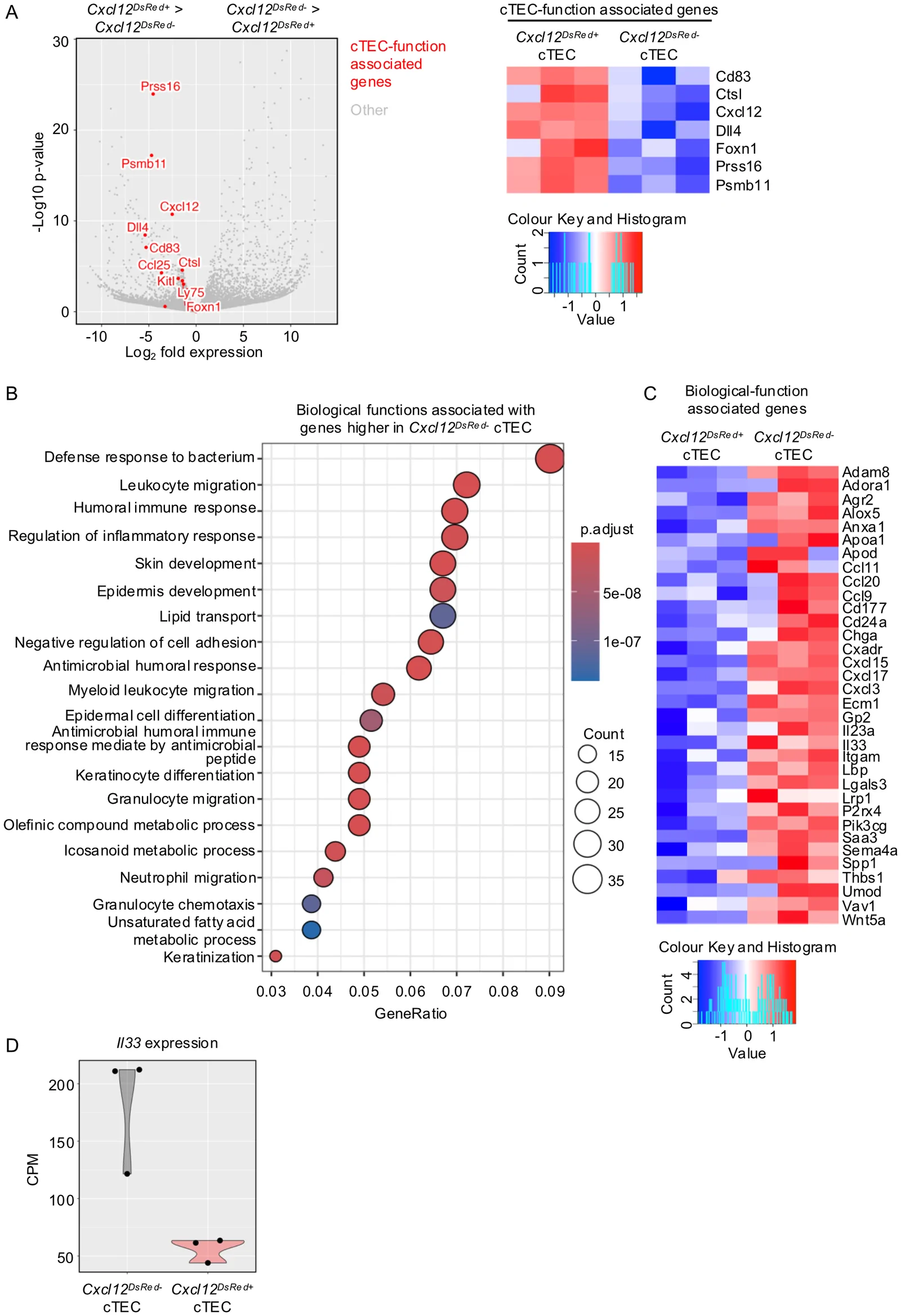
Loss of a thymopoiesis-inducing gene signature in atypical cTEC is accompanied by expression of multiple regulators of innate immune cells. *Cxcl12^DsRed^*^+^ and *Cxcl12^DsRed^*^−^ subsets of CD45^−^EpCAM1^+^UEA1^−^Ly51^+^ cTEC were FACS sorted from 10-wk-old adult *Cxcl12*^DsRed^ reporter mice, with experiments performed in triplicate to produce three independent biological replicates for each subset. **(A)** Volcano plot (left) of differentially expressed genes in FACS-sorted *Cxcl12^DsRed+^* (n=3) and *Cxcl12^DsRed−^* (n=3) cTEC. Significant cTEC-function associated genes (genes previously shown to be expressed by cTEC that regulate thymocyte development (15–20) are shown by red dots, with all other genes represented by gray dots. The right panel shows a heatmap of significantly differentially expressed genes associated with cTEC function for *Cxcl12^DsRed+^* and *Cxcl12^DsRed−^* cTEC. **(B)** Dot plot of Gene Ontology (GO) enrichment analysis of biological functions associated with genes higher in *Cxcl12*^DsRed−^ cTEC for the Top 20 pathways in the “clusterprofiler” database for the Biological Processes. **(C)** Heatmap of significantly differentially expressed genes identified by the GO analysis from **(B)**, focussing on the pathways associated with chemotaxis and migration, for *Cxcl1*2^DsRed+^ and *Cxcl12^DsRed−^* cTEC (34 genes). **(D)** Violin plot of *Il33* gene expression for *Cxcl12^DsRed+^* and *Cxcl12^DsRed−^* cTEC.

As atypical cTEC lack expression of genes associated with thymocyte development and selection, and to better understand their potential importance, we further analyzed the gene expression profile of these cells. Such analysis showed that genes differentially expressed by atypical cTEC but not typical cTEC were enriched for multiple biological processes, including defense responses to infection, and chemotaxis/migration, the latter including genes controlling innate immune cell migration e.g. granulocyte migration and chemotaxis, myeloid leukocyte migration, and neutrophil migration (**Figure 1B**). Further analysis of genes associated with these pathways revealed several chemokines primarily associated with immune cell migration to be expressed by atypical *Cxcl12^DsRed-^* but not typical *Cxcl12^DsRed+^* cTEC, including *Ccl11, Ccl20*, and *Ccl9* (**Figures 1B, C**). Surprisingly, we also identified selective expression of *Il33* in atypical *Cxcl12^DsRed-^* cTEC, a previously reported product of thymic mesenchyme which plays an essential role in regulating thymus recovery from acute injury (**Figures 1C, D**) (33–35). Collectively, these findings strengthen the idea that the adult cTEC compartment is heterogenous, and further suggests that rather than representing moribund cells, atypical cTEC that have lost the ability to influence thymocyte development acquire the expression of gene signatures previously linked to control of immune cell migration and thymus repair.

### Expression of IL33 by atypical cTEC is developmentally controlled

Given the detection of *Il33* gene expression within atypical cTEC isolated from adult mice, and its known importance in the control of intrathymic innate immune pathways (35), we crossed IL33-citrine reporter mice (*Il33^CIT^*) to *Cxcl12^DsRed^* reporter mice, using the subsequent *Cxcl12^DsRed^/Il33^CIT^* dual reporter mice to study the requirements for *Il33^CIT+^* cTEC development. Following enzymatic digestion, and as shown previously (31), we found that EpCAM1^+^Ly51^+^UEA-1^-^ cTEC from adult (10 week old) mice contained a mixture of *Cxcl12^DsRed+^* typical and *Cxcl12^DsRed-^* atypical cTEC (**Figure 2A**). Interestingly, cTEC from 10-week-old *Cxcl12^DsRed^/Il33^CIT^* dual reporter mice were also found to be heterogeneous for *Il33^CIT^* expression, with a readily detectable *Il33^CIT+^* population constituting approximately 25% of the total cTEC compartment (**Figure 2A**). By contrast to cTEC, few if any *Il33^CIT+^* cells were detectable within EpCAM1^+^Ly51^-^UEA-1^+^ mTEC (**Figure 2A**). To investigate the timing of appearance of *Il33^CIT+^* cTEC, we performed ontogenetic analysis of the cTEC compartment across the life-course in *Cxcl12^DsRed^/Il33^CIT^* mice. At neonatal stages (1–3 days post birth), the vast majority of cTEC were *Cxcl12^DsRed+^Il33^CIT-^* (**Figure 2B**). By 2 weeks of age, cTEC from *Cxcl12^DsRed^/IL33^CIT^* mice showed distinct heterogeneity in relation to *Cxcl12* and *Il33* expression. Here, alongside a dominant *Cxcl12^DsRed+^Il33^CIT-^* population, we also detected *Cxcl12^DsRed-^Il33^CIT-^* and *Cxcl12^DsRed+^Il33^CIT+^* subsets. Such populations were also detectable at 4 weeks of age, and by 6 weeks of age we saw the appearance of an additional *Cxcl12^DsRed-^Il33^CIT+^* cTEC subset, which was also detected at 10 weeks of age (**Figure 2B**). Collectively, these findings show that the adult cTEC compartment contains multiple distinct subsets that can be defined by *Cxcl12* and *Il33* expression. Furthermore, they show that induction of *Il33* expression within cTEC is a developmentally regulated process which appears first within typical *Cxcl12^DsRed+^* cells and then persists in a subset of atypical *Cxcl12^DsRed-^* cTEC.

**Figure 2:**
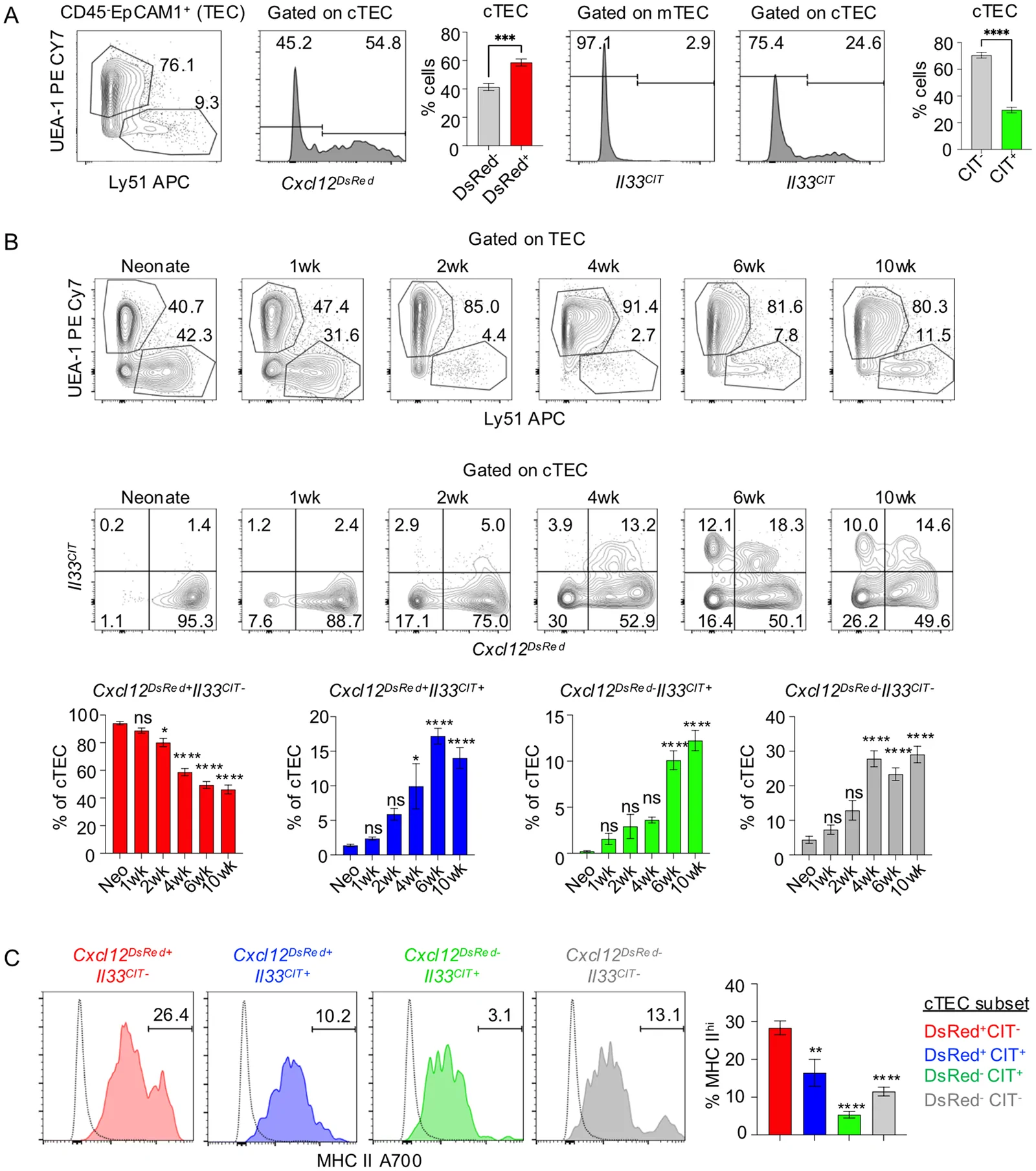
Atypical cTEC acquire IL33 in a developmentally controlled manner. **(A)** Expression of *Cxcl12^DsRed^* and *Il33^CIT^* in Ly51^+^UEA-1^-^ cTEC and UEA-1^+^Ly51^-^ mTEC from 10-week-old *Cxcl12^DsRed^Il33^CIT^* mice. **(B)** Analysis and quantitation of *Cxcl12^DsRed^* and *Il33^CIT^* expression in cTEC and mTEC at multiple stages of development. Statistical analysis of ontogeny for each age is compared to the neonate. **(C)** Analysis of MHC II expression by cTEC subsets at 10 weeks, the black line in histograms indicates staining levels on CD45^+^ thymocytes to serve as an internal negative control. Statistical analysis of each population is compared to *Cxcl12*^DsRed+^*Il33*^CIT-^. All stages were analyzed in at least 3 independent experiments, with minimum of n=5. For statistical analysis of **(A)**, a Mann–Whitney nonparametric test was carried out, and for **(B, C)**, One way ANOVA tests were carried out, with *p* values as follows: **p < 0.01, ***p < 0.001, ****p < 0.0001. Error bars represent mean ± SEM.

Since atypical cTEC show a downregulation of genes associated with antigen processing and presentation for positive selection (**Figure 1A**), we next examined cell surface expression of MHC II in the *Cxcl12^DsRed^/Il33^CIT^* adult cTEC populations described above. Flow cytometric analysis of digested 10-week-old thymus showed that while almost all cTEC continued to express MHC II, cells expressing the highest levels of MHC II were most abundant in the *Cxcl12^DsRed+^Il33^CIT-^* subset, with *Cxcl12^DsRed-^Il33^CIT+^* cTEC containing the lowest proportion of MHC II^hi^ cells (**Figure 2C**). Thus, loss of Foxn1-dependent *Cxcl12* expression and acquisition of *Il33* expression is accompanied by downregulation of MHC II expression in cTEC, paralleling the loss of genes associated with antigen processing and presentation for positive selection.

### *Il33^+^* atypical cTEC are generated from *Cxcl12^+^* typical cTEC

Our demonstration that cTEC heterogeneity emerges progressively across the life-course may suggest possible precursor-product relationships between the *Cxcl12^DsRed^/Il33^CIT^* cTEC subsets described above. To address this, we established reaggregate thymus organ cultures (RTOC) to directly assess the developmental potential of *Cxcl12^DsRed+^Il33^CIT-^* cTEC that dominate the cortex at neonatal stages. *Cxcl12^DsRed+^Il33^CIT-^* cTEC were purified from neonatal H-2^b^ C57Bl/6 *Cxcl12^DsRed^/Il33^CIT^* mice and mixed at a 1:10 ratio in RTOC with freshly disaggregated embryonic day 15 (E15) thymus lobes from H-2^d^ BALB/c mice (**Figure 3A**). As described previously with this approach (29, 36), the ‘spiked’ cell population of interest (i.e. *Cxcl12^DsRed+^Il33^CIT-^* cTEC) can be readily distinguished from ‘filler’ H-2^d+^ cells on the basis of their expression of H-2^b^ haplotype MHC II molecules. After 7 days, RTOC were enzymatically digested and Ly51^+^UEA-1^-^ cTEC were analyzed for expression of *Cxcl12^DsRed^* and *Il33^CIT^*. In contrast to RTOC established with H-2^d^ BALB/c filler cells alone, RTOC initiated with a spiked population of C57BL/6-derived *Cxcl12^DsRed+^Il33^CIT-^* cTEC contained a readily detectable I-A^b^ subset within Ly51^+^ cTEC (**Figure 3B**). Importantly, such spike-derived I-A^b+^ Ly51^+^ cTEC contained *Cxcl12^DsRed-^* cells, which further contained *Il33^CIT-^* and *Il33^CIT+^* cells (**Figure 3C**). While it remains unclear whether *Cxcl12^DsRed+^Il33^CIT-^* cells downregulate *Cxcl12^DsRed^* before or after acquiring *Il33^CIT^* expression, these data demonstrate that neonatal *Cxcl12^DsRed+^Il33^CIT-^* cTEC can generate multiple subsets of *Cxcl12^DsRed-^* cTEC, including those expressing *Il33*, which identifies at least one cellular source for this subset of atypical cTEC.

**Figure 3:**
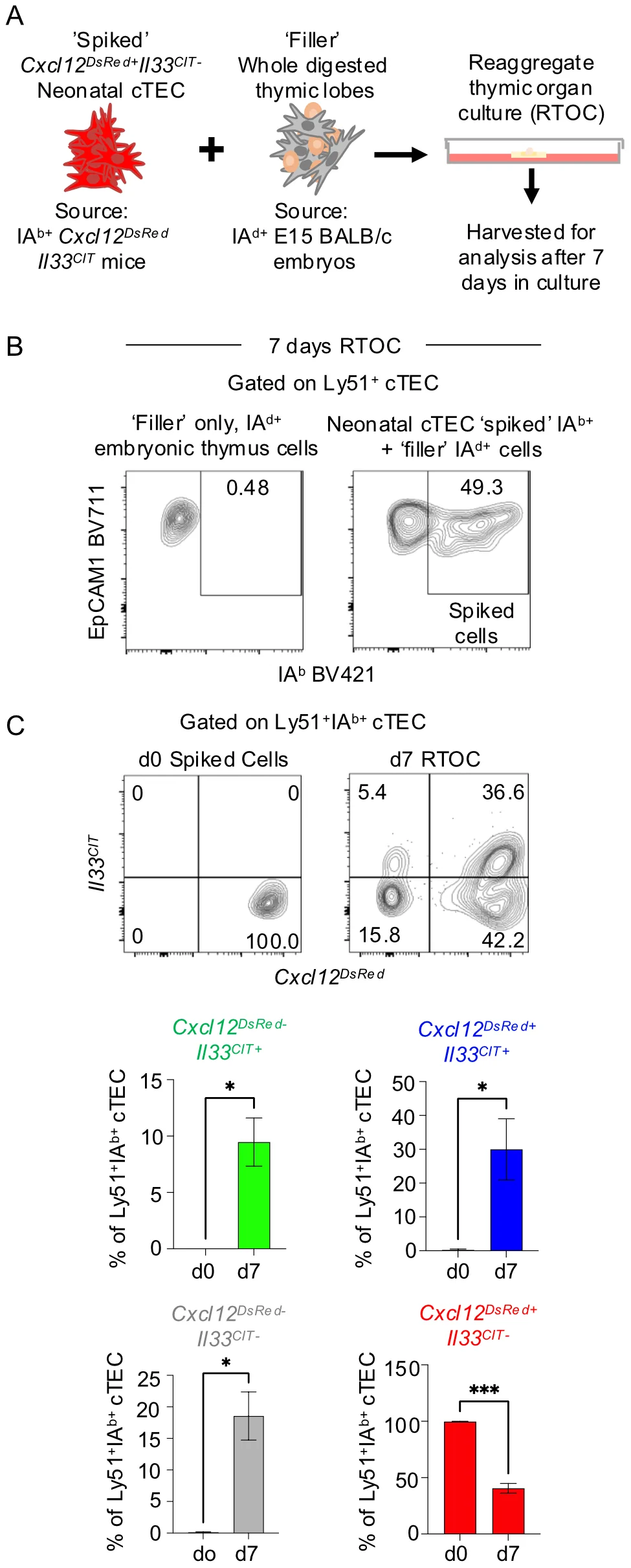
*Il33^CIT+^* atypical cTEC are generated from *Cxcl12^DsRed+^* typical cTECs. **(A)** Reaggregate thymic organ culture (RTOC) experimental scheme to assess the developmental potential of purified neonatal *Cxcl12^DsRed+^Il33^CIT-^* cTEC. **(B)** Analysis of digested RTOC after 7 days culture, formed from RTOC made from either E15 BALB/c IA^d+^ cells alone (i.e. filler cells, left panel), or containing purified *Cxcl12^DsRed+^Il33^CIT-^* cTEC from *Cxcl12^DsRed^Il33^CIT^* IA^b+^ mice (i.e. spiked cells, right panel). Plots shown are following pre-gating on EpCAM1^+^Ly51^+^UEA1^-^ cells to identify total cTEC, where spiked cells were identified by IA^b^ expression, with gates set on RTOC containing IA^d+^ cells alone (left panel) to distinguish positive and negative levels of IA^b^ staining. **(C)** Analysis of *Cxcl12^DsRed^* and *Il33^CIT^* expression by FACS-sorted IA^b+^ cTEC at the initiation of RTOC culture (day 0) and following 7 days of culture. Quantitation of *Cxcl12^DsRed^* and *Il33^CIT^* populations at initiation of culture (d0) and day 7 (d7) of culture. A Mann–Whitney nonparametric test was carried out, and the *p* values are as follows: **p < 0.01, ***p < 0.001, ****p < 0.0001. Error bars represent mean ± SEM. Plots are representative of individual 4 experiments.

### Progression beyond the Pre-TCR mediated checkpoint of thymocyte development controls *Il33*^+^ atypical cTEC development

Given the importance of thymocyte-stromal cell crosstalk in the formation and functioning of TEC microenvironments (37, 38), we next examined how specific stages of thymocyte development might influence the *Il33^CIT^*^+^ atypical cTEC subset. For maturation of CD4^-^CD8^-^ thymocytes, a key checkpoint occurs at the CD4^-^CD8^-^CD25^+^CD44^-^ double negative 3 (DN3) stage which is controlled by signaling through the pre-TCR, which consists of CD3, preTα and TCRβ proteins (39, 40). As thymocyte maturation in *Rag2*^-/-^ mice is arrested at the DN3 stage due to a lack of TCRβ rearrangement (41), we assessed the importance of progression beyond the pre-TCR mediated checkpoint in thymocyte development by crossing *Rag2^-/-^* mice with *Cxcl12^DsRed^Il33^CIT^* mice. As previously reported (42) compared to control (*Rag2^+/-^) Cxcl12^DsRed^Il33^CIT^* mice, UEA-1^+^ mTEC were largely absent from *Rag2^-/-^Cxcl12^DsRed^Il33^CIT^* mice, while Ly51^+^UEA-1^-^ cTEC were readily detectable (**Figure 4A**). Importantly, analysis of this cTEC compartment in the absence of *Rag2* expression showed marked differences. For example, while the proportion of *Cxcl12^DsRed+^Il33^CIT-^* cTEC was increased and *Cxcl12^DsRed+^Il33^CIT+^* cTEC were comparable, both *Cxcl12^DsRed-^Il33^CIT+^* and *Cxcl12^DsRed-^Il33^CIT-^* cTEC were significantly reduced (**Figures 4B, C**). Moreover, analysis of MHC II expression showed significantly more cTEC expressed high levels of MHC II in *Rag2*^-/-^ mice compared to *Rag2*^+/-^ controls (**Figures 4B, C**).

**Figure 4:**
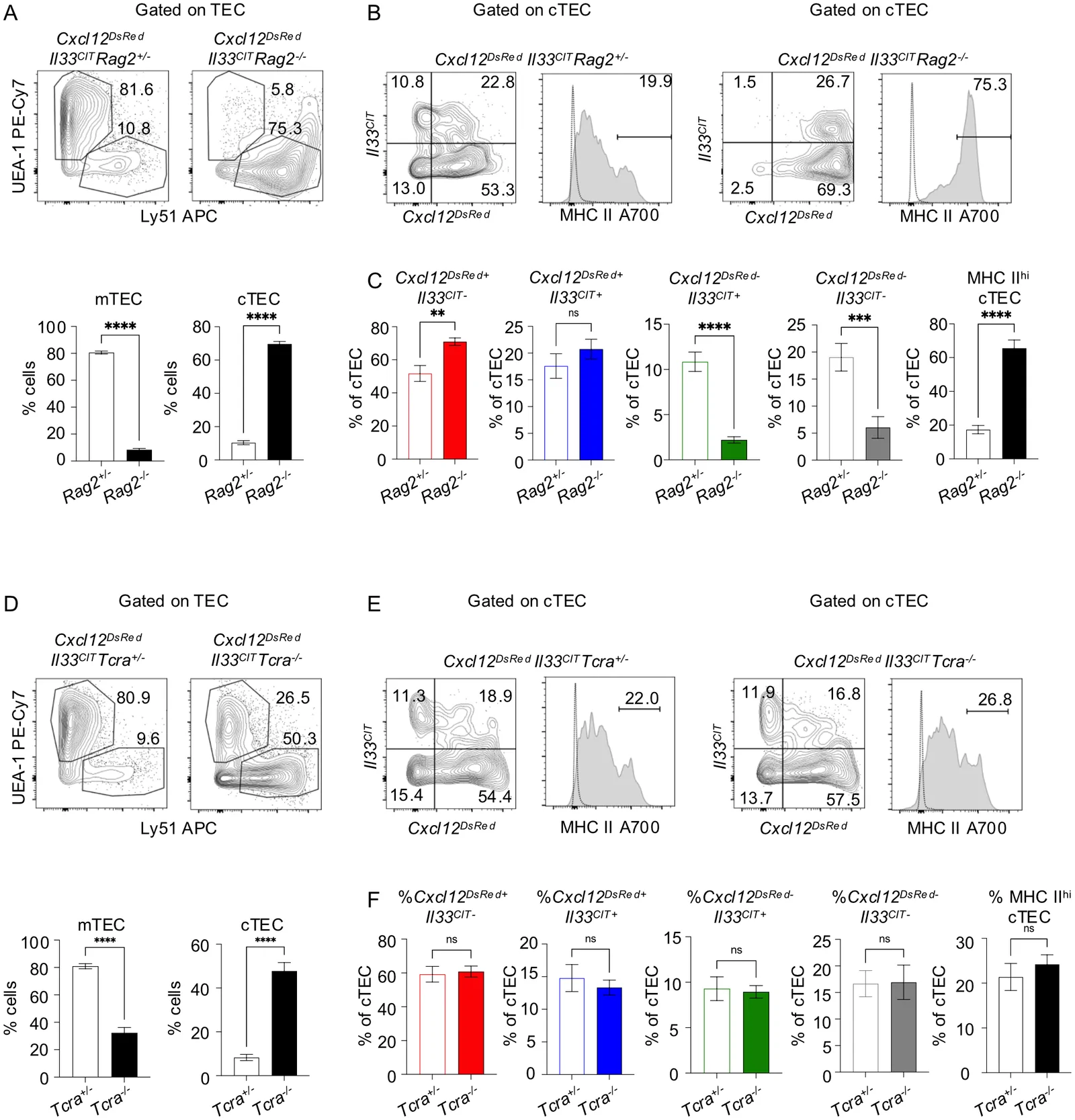
Progression beyond the Pre-TCR checkpoint of thymocyte development controls *Il33^CIT+^* atypical cTEC development. **(A)** Identification of cTEC (UEA-1^-^Ly51^+^) and mTEC (UEA-1^+^Ly51^-^) in 10-week-old *Cxcl12^DsRed^Il33^CIT^Rag2^+/-^* or *Cxcl12^DsRed^Il33^CIT^Rag2^-/-^* mice. Panels **(B, C)** show analysis and quantitation of *Il33^CIT^* and *Cxcl12^DsRed^* expression in cTEC from *Cxcl12^DsRed^Il33^CIT^Rag2^+/-^* and *Cxcl12^DsRed^Il33^CIT^Rag2^-/-^* mice, alongside expression of MHC II. The black line in histograms shows staining levels on CD45^+^ thymocytes that serves as an internal negative control. Panels **(D-F)** show the same analysis of cTEC in 10-week-old *Cxcl12^DsRed^Il33^CIT^Tcra^+/-^* and *Cxcl12^DsRed^ll33^CIT^Tcra^-/-^* mice. Data are from at least 3 independent experiments, with minimum of n=6. Mann–Whitney nonparametric test was carried out, and the *p* values are as follows: **p < 0.01, ***p < 0.001, ****p < 0.0001. Error bars represent mean ± SEM.

To further examine the impact of thymocyte crosstalk on cTEC heterogeneity, we next crossed *Cxcl12^DsRed^Il33^CIT^* mice with *Tcra^-/-^* mice, where thymocytes are blocked at the CD4^+^CD8^+^ stage due to lack of αβTCR expression (43, 44). In contrast to analysis of cTEC in the absence of *Rag2* expression, we saw no differences in proportions of *Cxcl12^DsRed^*/*Il33^CIT^* subsets in the absence of *Tcra* expression (**Figures 4D–F**). Collectively, these data demonstrate a stage-specific requirement for thymocyte crosstalk in the generation of cTEC heterogeneity, with the emergence of both *Il33^CIT+^* and *Il33^CIT-^* subsets of *Cxcl12^DsRed-^* cTEC requiring signals that arise following progression beyond the pre-TCR mediated checkpoint in thymocyte development.

## Discussion

The thymic cortex is well known as a key site for multiple stages in thymocyte development (6). Here, the further maturation of immature CD4^-^CD8^-^ and CD4^+^CD8^+^ thymocytes is controlled by cTEC through their expression of a panel of Foxn1-dependent genes including *Cxcl12, Dll4*, and *Psmb11*. Importantly, while provision of signals for thymopoiesis is a key function of the cortex, our recent identification of an atypical cTEC subset that lacks this thymopoietic-inducing signature (31) raises the possibility that the cortex may play additional roles in thymus function. Here, we examined the properties and developmental requirements of atypical cTEC and show they represent a thymocyte-controlled population defined by a gene expression profile that may link them to roles distinct from supporting thymopoiesis.

An important aspect of understanding the cTEC heterogeneity described here is the possible precursor-product relationships that exist between distinct *Cxcl12^DsRed^/Il33^CIT^* cTEC subsets. Using RTOC systems to track the developmental potential of a single cohort of *Cxcl12^DsRed+^Il33^CIT-^* cTEC, we saw that these cells were able to generate *Cxcl12^DsRed-^* cTEC within a 7-day culture period. Moreover, some *Cxcl12^DsRed-^* cTEC had also acquired *Il33^CIT^* expression. Thus, atypical *Il33^CIT+^* cTEC can arise from typical *Cxcl12^DsRed+^* cTEC, which identifies at least one source of these cells. However, it is currently unclear whether *Cxcl12^DsRed-^Il33^CIT+^* cTEC are generated directly from *Cxcl12^DsRed+^Il33^CIT+^* intermediates or are generated after the downregulation of *Cxcl12* expression from *Cxcl12^DsRed-^Il33^CIT-^* cells. We also performed ontogenetic analysis to aid in understanding these possible cTEC precursor-product relationships. Here, while cTEC at neonatal stages were defined by a homogeneous *Cxcl12^DsRed+^Il33^CIT-^* phenotype, at 2 weeks of age we saw the appearance of both *Cxcl12^DsRed+^Il33^CIT+^* and *Cxcl12^DsRed-^Il33^CIT-^* prior to the appearance of *Cxcl12^DsRed-^Il33^CIT+^*. Given the simultaneous emergence of *Cxcl12^DsRed+^Il33^CIT+^* and *Cxcl12^DsRed-^Il33^CIT-^* cTEC at this early life stage, it remains unclear whether either, or both, can act as sources of *Cxcl12^DsRed-^Il33^CIT+^* atypical cTEC. Further work is required to gain a full understanding of the precursor-product relationships that determine cTEC heterogeneity.

Of key importance is to gain an understanding of the functional relevance of adult cTEC heterogeneity, and atypical IL33-expressing cTEC in particular. Our finding that adult cTEC act as a source of this alarmin may be important in relation to mechanisms of thymus regeneration following damage (35). Previously, we identified thymic mesenchyme cells as a dominant source of IL33, which plays a key role in controlling a type 2 innate immune pathway via the activation of ILC2 (33). By identifying atypical cTEC as an additional source of IL33, our current findings suggest a scenario in which both cTEC and mesenchyme may influence thymus regeneration via IL33 production. Functional assessment of the relative contributions of cTEC and mesenchyme will require analysis of thymus regeneration in models involving cell-type specific deletion of IL33. In addition to IL33, RNA bulk sequencing analysis also demonstrated the selective presence of several regulators of innate immune cell migration within *Cxcl12^DsRed-^* atypical cTEC, including *Ccl11*, an important chemokine that through interactions with CCR3 controls the intrathymic availability of eosinophils (33). Given the involvement of both eosinophils and IL33 in thymus regeneration, this may also indicate the potential for atypical cTEC in thymic regeneration programs. Further experiments, such as conditional deletion of CCL11 in cTEC, would be required to demonstrate direct involvement of atypical cTEC in the control of thymic eosinophils. Collectively, these findings suggest that while atypical cTEC have lost the ability to influence at least some aspects of thymocyte development, they have gained the ability to influence innate immune thymic cells, some of which, namely eosinophils, play an essential role in thymus repair.

Finally, it is interesting to note that the generation of atypical IL33^+^ cTEC requires expression of the pre-TCR, but not αβTCR, in developing thymocytes, which is evidenced by the paucity of IL33^+^ atypical cTEC in *Rag2^-/-^Il33^CIT^* mice. Importantly however, it is not clear from these experiments whether this defect in cTEC development is caused by a lack of pre-TCR signaling in thymocytes, which may directly provide signals that then influence cTEC development. Indeed, *Rag2^-/-^* mice also display profound defects in thymus organization, which could also influence the development of the cTEC compartment. While further work is required to examine these scenarios, our data supports a process in which progression beyond the pre-TCR checkpoint can influence cTEC development. Interestingly, these findings are somewhat distinct from the requirements mTEC maturation, where CD4^+^CD8^+^ thymocytes are required to generate single positive CD4^+^ and CD8^+^ progeny (43, 44). While the signals derived from cTEC-thymocyte crosstalk that shape the thymic cortex are not known, these findings reinforce the notion that the epithelial cells within the two major anatomical compartments of the thymus, cortex and medulla, are both shaped from thymocytes at distinct stages of their own development.

## Materials and methods

### Mice

Mice on a C57BL/6 background were obtained from The Jackson Laboratory and used at 8–10 weeks of age unless otherwise specified. *Cxcl12^DsRed^* knock-in mice (JAX stock no. 022458) were crossed with *Il33*-citrine (*Il33^CIT^*) reporter mice (45) and used either alone or further crossed with *Tcra*^−/−^ (JAX stock no. 002116) or *Rag2*^−/−^ (JAX stock no. 008449) mice. Heterozygous littermates were used as controls for experiments involving *Tcra*^−/−^ and *Rag2*^−/−^ strains. Wildtype BALB/c mice (H-2^d^ haplotype) were used to generate timed matings, with the day of detection of a vaginal plug designated day 0 of gestation, and embryos were used at embryonic day E15 of gestation. In all experiments, a mixture of male and female mice was used. All mouse husbandry, housing, and experimental procedures were carried out at the Biomedical Services Unit, University of Birmingham, in accordance with protocols approved by the local Ethical Review Panel and in compliance with UK Home Office regulations (Animal Project License No. PP7518148).

### Preparation of thymic epithelial cells for flow cytometry

For thymic epithelial cell (TEC) analysis, thymic lobes at the indicated ages were placed in 1ml of RPMI 1640 medium containing 10% Fetal Calf Serum, Collagenase/Dispase (2.5 mg/mL; Roche) and DNase I (250µg/mL; Roche). Thymic lobes were cut into 1mm^3^ pieces using sharp scissors and then incubated at 37°C for 60 minutes, with samples being mechanically agitated every 15 minutes using a 1ml pipette to aid disaggregation. The resultant single cell suspension was centrifuged, supernatant was discarded, and samples were resuspended in 1ml of MACS buffer (Miltenyi Biotec). CD45^−^ cells were then subsequently enriched by depletion of CD45^+^ cells using anti-CD45 magnetic beads (Miltenyi Biotec) and LS columns according to manufacturer’s guidelines (Miltenyi Biotec). The following antibodies/reagents were used for flow cytometric analysis: anti-CD45 APC 780 (clone 30-F1, eBioscience), anti-EpCAM1 BV711 (clone G8.8, eBioscience), MHC II A700/BV421 (clone M5/114.15.2, eBioscience), Ly51 APC (clone 6C3, Biolegend), CD80 BV605 (clone 16-10A1, Biolegend), biotinylated UEA-1 (Vector Laboratories), and streptavidin PE Cy7 (eBioscience). All samples were analyzed using an LSR Fortessa (Becton Dickinson) and data analysis was performed using FlowJo V10 (Becton Dickinson).

### Bulk RNAseq

RNA sequencing data were reanalyzed from data obtained from our previously published dataset (GSE205940) (31). Briefly, RNA samples were extracted using the Qiagen RNeasy kit. Libraries were prepared using the SMARTer Ultra Low Input RNA Kit for Sequencing as per the manufacturer’s instructions and sequenced on an Illumina NovaSeq platform. Reads were trimmed for adapter contamination using Trimmomatic (version 0.36) and aligned to the mm10 mouse genome using STAR (version 2.7.3a) (46, 47). Reads were assigned to genes using HTSeq (version 0.12.4) with the option “intersection-nonempty” (48). Differentially expressed genes were identified using edgeR (false discovery rate < 0.05) (49). For gene ontology over-representation analysis, a more stringent threshold of FDR < 0.01 combined with an absolute log2 fold change > 1 was applied to reduce the inclusion of genes with statistically significant but biologically trivial expression differences. Gene ontology analysis was performed using clusterProfiler (50), and analyses were performed separately for genes higher in *Cxcl12^DsRed^*^-^ and *Cxcl12^DsRed+^* cTEC.

### Reaggregate thymus organ cultures (RTOC)

Reaggregate thymic organ cultures (RTOCs) were established as previously described (51). Briefly, freshly dissected thymic lobes from 2-day old neonatal *Cxcl12^DsRed^Il33^CIT^* mice were digested in the same way as adult thymus described above, using the enzymes Collagenase/Dispase (2.5 mg/mL; Roche) and DNase I (250µg/mL; Roche). Following dissagregation, neonatal thymic suspensions were enriched for CD45^-^ stromal cells using anti-CD45 magnetic beads and LS columns (Miltenyi Biotec). Stromal enriched preparations were stained using the following antibodies/reagents: anti-CD45 APC 780, anti-EpCAM1 BV711, anti-Ly51–647 and biotinylated UEA-1, followed by Streptavidin PECy7. CD45^−^EpCAM^+^Ly51^+^UEA-1^−^ cTEC were then FACS sorted using a FACSAria Fusion 1 cell sorter (Becton Dickinson), typically to greater than 99% purity (**Figure 3**). These cells are referred to as the ‘spike’ population for RTOC formation. Separately, freshly dissected E15 BALB/c thymic lobes were placed in a solution of 0.25% trypsin/0.02% EDTA (Sigma Aldrich), incubated at 37°C for 5–10 minutes and gently pipetted until a single cell suspension was obtained. Cells were then transferred to medium containing 10% FCS to neutralize trypsin, and then centrifuged. Such cells are referred to as the ‘filler’ population. For RTOC formation, ‘spiked’ cTEC were combined with the E15 BALB/c ‘filler’ thymic suspension at a 1:10 ratio. Typically, 50,000 ‘spiked’ FACS-sorted cTEC were combined with 500,000 E15 BALB/c ‘filler’ cells. Cell mixtures were then combined and centrifuged, with the resulting pellet placed onto the surface of an 0.8µm Nucleopore filter supported by a sponge square (51). Control RTOC were generated using ‘filler’ E15 BALB/c thymus tissue alone. After 7 days, RTOC were dissociated using 0.25% trypsin/0.02% EDTA (Sigma Aldrich) in the same way as for fresh E15 thymic lobes, and stained using the following antibodies/reagents: anti-CD45 APC 780 (eBioscience), anti-EpCAM1 BV711 (eBioscience), anti-IA^b^ BV421 (clone AF6-120.1, Becton Dickinson), Ly51 APC (Biolegend), biotinylated UEA-1 (Vector Laboratories), and Streptavidin PE Cy7 (eBioscience).

## Data Availability

Bulk RNA sequencing data from atypical and typical cTEC subsets are available at the Gene Expression Omnibus (GEO; accession number GSE205940 https://www.ncbi.nlm.nih.gov/geo/query/acc.cgi?acc=GSE205940).
